# Memristive LIF Spiking Neuron Model and Its Application in Morse Code

**DOI:** 10.3389/fnins.2022.853010

**Published:** 2022-04-07

**Authors:** Xiaoyan Fang, Derong Liu, Shukai Duan, Lidan Wang

**Affiliations:** ^1^College of Artificial Intelligence, Southwest University, Chongqing, China; ^2^Department of Electrical and Computer Engineering, University of Illinois at Chicago, Chicago, IL, United States

**Keywords:** LIF, MLIF, memristor, spiking patterns, neuron, Morse code

## Abstract

The leaky integrate-and-fire (LIF) spiking model can successively mimic the firing patterns and information propagation of a biological neuron. It has been applied in neural networks, cognitive computing, and brain-inspired computing. Due to the resistance variability and the natural storage capacity of the memristor, the LIF spiking model with a memristor (MLIF) is presented in this article to simulate the function and working mode of neurons in biological systems. First, the comparison between the MLIF spiking model and the LIF spiking model is conducted. Second, it is experimentally shown that a single memristor could mimic the function of the integration and filtering of the dendrite and emulate the function of the integration and firing of the soma. Finally, the feasibility of the proposed MLIF spiking model is verified by the generation and recognition of Morse code. The experimental results indicate that the presented MLIF model efficiently performs good biological frequency adaptation, high firing frequency, and rich spiking patterns. A memristor can be used as the dendrite and the soma, and the MLIF spiking model can emulate the axon. The constructed single neuron can efficiently complete the generation and propagation of firing patterns.

## 1. Introduction

Neurons, as the dual-role of the function units of the perceiving-conducting stimulus and the information processing, can carry out particular tasks of sensory, motor, neural responses, and cognition (Hirokawa et al., 2019), and so on. Many neuron models emerged (Hodgkin and Huxley, 1952a; FitzHugh, 1961; Morris and Lecar, 1981; Bernander et al., 1994; Izhikevich, 2003) to mimic the functions of a biological neuron, especially the LIF spiking model. It is a simpLIFied and much easier model for hardware implementation and large-scale integration (Slepova and Zhilenkov, 2018). The primary purpose of an artificial neuron is to mimic the functions of biological neurons in an energy effectiveness and scalability way. The typical LIF model consists of a capacitor and a resistor. The external stimulus is applied to the LIF model until a threshold is reached, and then the action potential is produced (Han and Meyyappan, 2018). It is widely applied in bioinspired and brain-inspired neuromorphic information processing systems (Belkaid and Krichmar, 2020; Neves and Timme, 2020; Yang and Kim, 2020; Doutsi et al., 2021). Although the LIF model can reproduce the firing behaviors of neurons after each activation, the previous pulse cannot be retained, and the biological spiking frequency adaptability does not perform very well. To solve these deficiencies, we need to find a new device to promote the LIF neuron model. A memristor is a potential element to emulate the function and behavior of a biological synapse or neuron (Hu et al., 2016; Choi et al., 2018; Chen et al., 2019; Greenberg-Toledo et al., 2019; Xia and Yang, 2019; Wang et al., 2020; Shi and Zeng, 2021) gets a lot of attention. The non-volatile memristor modulates its conductance due to ion motion, similar to the phenomena in biological neurons and synapses. Therefore, these advantages enable the memristor to become an inevitable choice as a building block between artificial neural networks and biological neural networks. The LIF oscillatory neuron with a memristor is used to perform threshold and firing functions (Jiang and Hu, 2021), and the LIF neuron with a threshold switching memristor realizes the firing behavior is driven by the threshold (Dev et al., 2020). A flexible memristor is integrated into the LIF neuron, generates four firing patterns, and implements the transformation between analog signals and spiking signals (Zhu et al., 2021). The TSM (threshold switching memristor) LIF neuron circuit experimentally performs the integrate and fire behaviors (Xu et al., 2022). The diffusive memristor LIF neuron model mimics neuron integration, leakage, spatiotemporal, and firing activities (Yang et al., 2020). The LIF neuron combined with a TSM can show the “leaky-integrate-fire” function and low power consumption (Lu et al., 2020).

Even though the LIF neuron model with a memristor had achieved lots of progress in emulating biological neurons, the implementation of retaining the previous pulse and performing the biological spiking frequency adaptability has not been yet explored in the MLIF neuron model. In our work, we first experimentally implement the MLIF neuron model. The memristor exhibits non-volatile behavior to “remember” the previous pulses by applying a series of pulses. In addition, the biological spiking frequency adaptability performs very well by combining the LIF neuron with a memristor. Furthermore, an individual neuron model formed by memristors is presented, and the distortionless transmission of the action potential is realized.

The primary work is to construct the memristive leaky integrate-and-fire spiking model after integrating a memristor to the LIF spiking model. In section 2, the LIF spiking model will be introduced to analyze it. In section 3, the MLIF spiking model is constructed. When the distinct stimuli act on the LIF and MLIF spiking models, the MLIF spiking model performs good biological adaptation, high firing frequency, and rich firing patterns in section 4. The memristor experimentally simulates the functions of synapse, dendrite, and soma, and an individual neuron is entirely constructed by memristors. It successfully reproduces the firing patterns and vividly emulates the information transmission of a biological neuron in section 5. Finally, the proposed model is further verified by the generation and recognition of Morse code in section 6. Section 7 is the conclusion of the article.

## 2. The LIF Spiking Neuron Model

**Figure 1:**
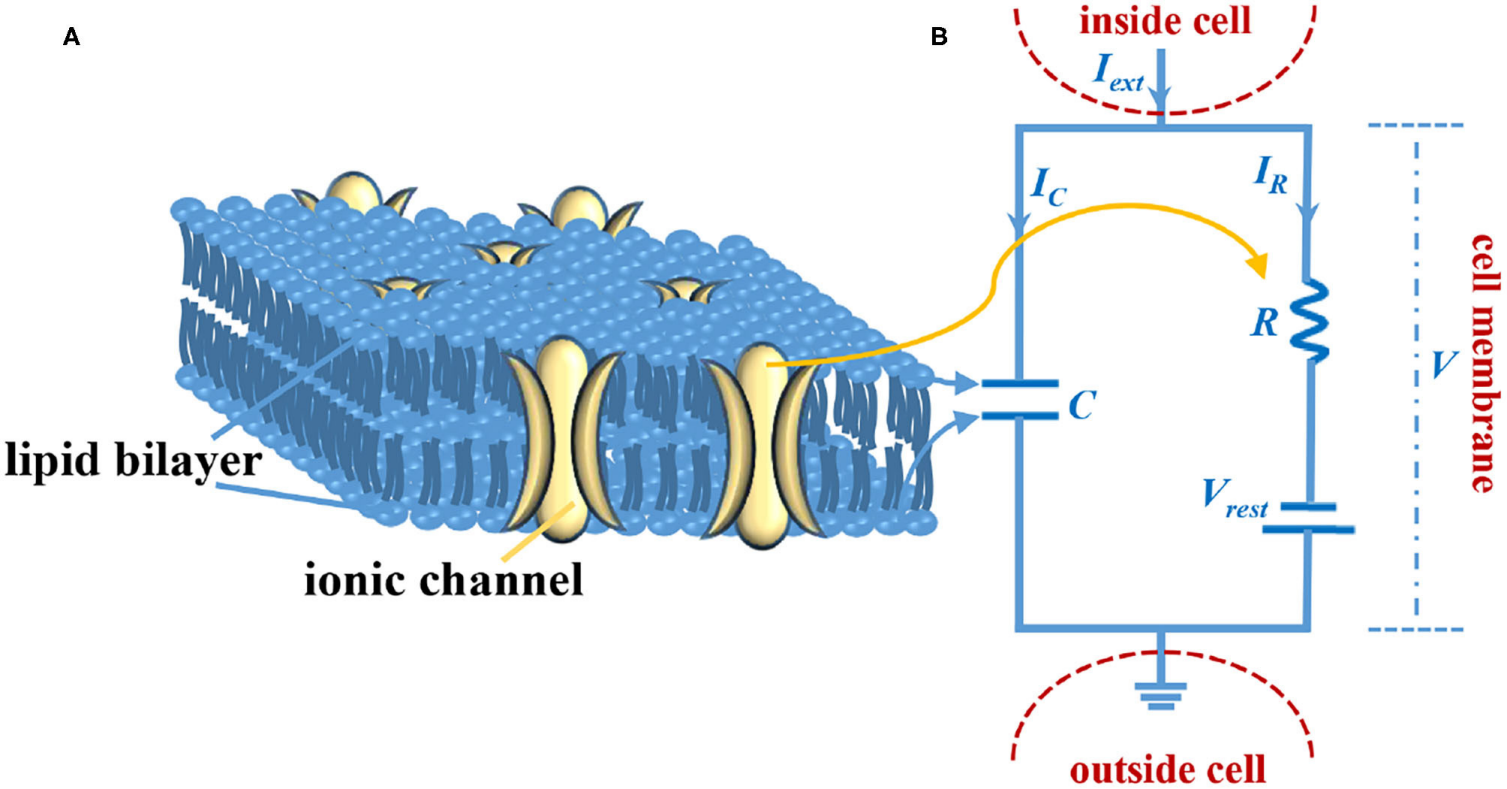
The LIF circuit model of the axon membrane. **(A)** The sketch of the cell membrane. **(B)** The circuit model of the cell membrane.

A. L. Hodgkin and A. F. Huxley detailed the generation mechanism of the action potential through carrying out many electrophysiological experiments on the squid giant axon. Meanwhile, they proposed the membrane electrical circuit to mimic the electrophysiological behaviors of the biological cell membrane (Hodgkin and Huxley, 1952a). The Hodgkin-Huxley (HH) membrane circuit can precisely describe the main characteristics of the axon membrane (Hodgkin and Huxley, 1952a,b,c,d); however, the equation calculation of the HH model is complex, and the large-scale neural networks are hard to construct. The LIF spiking circuit model is put forward to simpLIFy the HH model, which is closer to the real biological neuron, as shown in Figure 1 (Teka et al., 2014).

The cell membrane consists of the lipid bilayer and the ionic channel (Figure 1A). The lipid bilayer can be represented by a capacitor, and the ionic channel can be characterized by a resistor (Figure 1B). *I*_*ext*_ is external stimulus, *C* is the membrane capacitor, *R* is the membrane resistor (leaky resistor), *V*_*rest*_ is the resting voltage, *V*- *V*_*rest*_ is the resistive voltage, *I*_*C*_ is the current that passes through membrane capacitor, *I*_*R*_ is the current that passes through the membrane resistor, and *V* is the membrane voltage. Current passes through the membrane capacitor:


(1) $$\begin{array}{r} q=CV \end{array}$$

(2) $$\begin{array}{r} I_{C}=dq/dt=CdV/dt \end{array}$$

Current passes through the membrane resistor:


(3) $$\begin{array}{r} I_{R}=\left(V-V_{rest}\right)/R \end{array}$$

According to Kirchhoff's current law:


(4) $$\begin{array}{r} I_{ext}=I_{C}+I_{R} \end{array}$$

The time constant:


(5) $$\begin{array}{r} \tau =RC \end{array}$$

The differential equation of the LIF model, which represents the leaky integration process:


(6) $$\begin{array}{r} dt=-\left(V-V_{rest}\right)+RI_{ext} \end{array}$$

Using the finite differential method to solve (6) and compute the membrane potential at a time step of duration Δ*t*:


(7) $$\begin{array}{r} V\left(t+\Delta t\right)-V\left(t\right)=\Delta t/\tau \left(-V\left(t\right)+V_{rest}+RI_{ext}\right) \end{array}$$

The spiking generation process of the LIF circuit model: when *V*(*t*) reaches a certain threshold *V*_*th*_, the LIF model produces a spike artificially by setting *V*(*t*) = 20*mV*, and then *V*(*t*+Δ*t*) resets to be −80*mV* immediately.

## 3. The Memristive LIF (MLIF) Spiking Neuron Model

**Figure 2:**
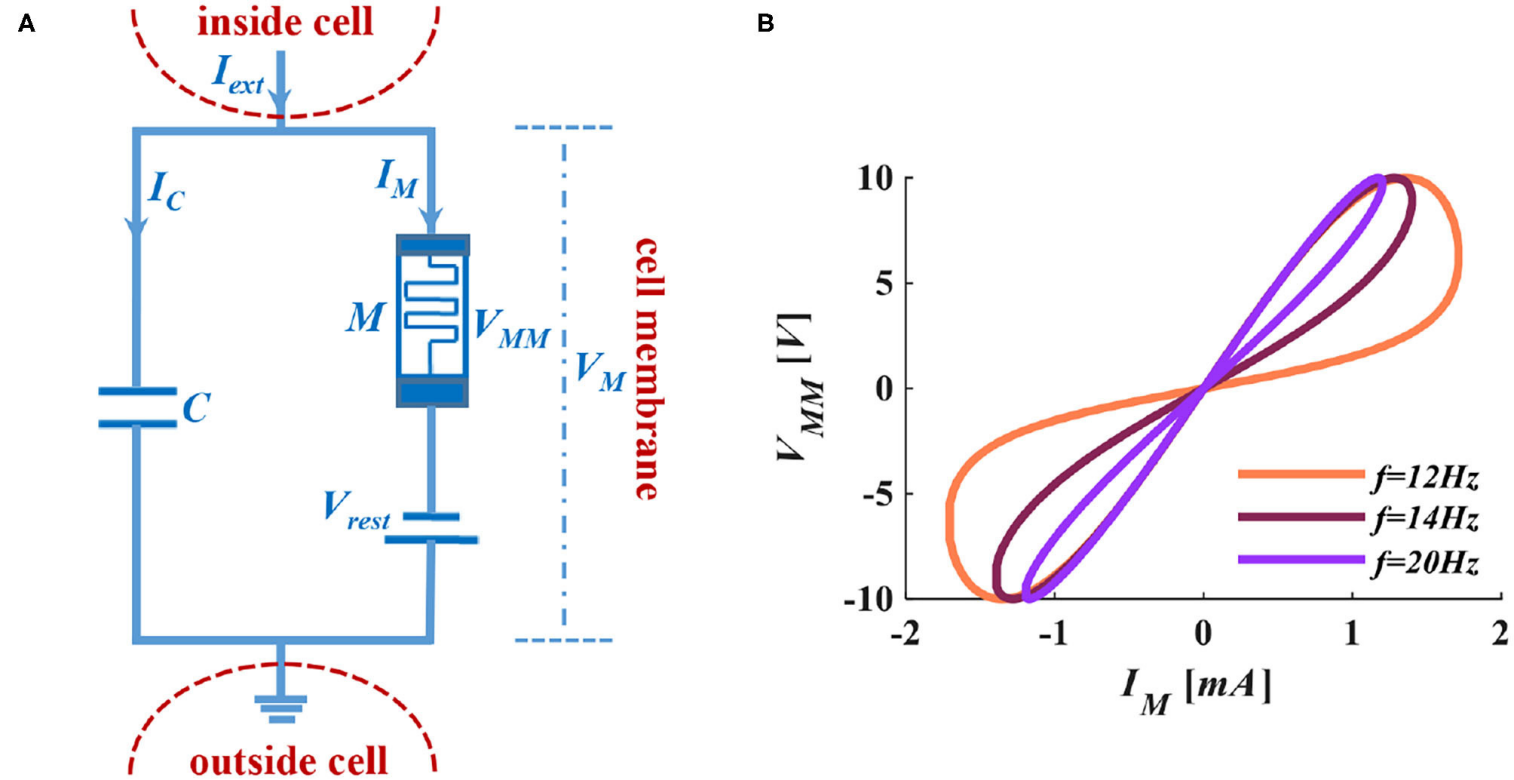
The MLIF circuit model and the I-V curve of the memristor. **(A)** MC membrane circuit of the MLIF model. **(B)** The pinched hysteresis curve and frequency characteristics of ion channel memristor.

Izhikevich analyzed and compared the advantages and disadvantages of the typical spiking neuron models (Izhikevich, 2004). Considering the LIF spiking model has no memory of the previous spike (Izhikevich, 2003) and the memory advantage of the memristor (can “remember” the charges pass through itself, and it is called non-volatile characteristics), we introduce a memristor to the LIF spiking model, as shown in Figure 2A.

When we apply a sinusoidal voltage to the ion channel memristor, it performs a zero-crossing pinched hysteresis curve. When we adjust the voltage frequency to 100*Hz*, the electrical characteristics of the memristor are close to a straight line. The memristor performs the feature of pure resistance (Adhikari et al., 2013). In Figure 2B, the distribution of the curve is in the first and third quadrants, which indicates that the device is passive. The curve has two prominent switching states and keeps a memristance constant without a power supply. It shows that the device is non-volatile.

In the MLIF membrane circuit, the τ is not a constant anymore, and it becomes a function of time. Therefore, τ = *RC* is transformed into τ_*M*_(*t*) = *M*(*t*)*C*. The memristor *M*(*t*) is divided into charge-controlled memristor and flux-controlled memristor, and they are the functions of time. According to *q* = *CV*, we get *q*(*t*) = *C*(*t*)*V*_*M*_ (*V*_*M*_ is the membrane voltage of the MLIF model, as shown in Figure 2A), thereby we can rewrite *C* as *C*(*t*) = *q*(*t*)/*V*_*M*_, and τ_*M*_(*t*) = *M*(*t*)*q*(*t*)/*V*_*M*_. The charge or discharge time of the capacitor always relates to the accumulation of charges.

The charge-controlled memristor (Wang et al., 2012b):


(8) $$\begin{array}{l} M(q(t))=\\ \left\{ \begin{array}{l} \;20000\;q(t)<-0.5\times 10^{-4}\\ 10^{4}+(-1.99)\times 10^{8}\times q(t)q(t)\geq -0.5\times 10^{-4}\;and\;q(t)<0.5\times 10^{-4}\\ \;100\;q(t))\geq 0.5\times 10^{-4} \end{array}\right. \end{array}$$

And then, we get:


(9) $$\begin{array}{l} \tau_{M}(t)=\\ \left\{ \begin{array}{l} \;20000q(t)/V_{M}\;\varphi (t)<-0.75\\ 10^{4}q(t)/V_{M}+(-1.99)\times 10^{8}\times q{\left(t\right)}^{2}/V_{M}\varphi \left(t\right)\geq -0.75and\varphi \left(t\right)<0.25\\ \;100q\left(t\right)/V_{M}\;\varphi \left(t\right))\geq 0.25 \end{array}\right. \end{array}$$

The relationship between charge and flux:


(10) $$\begin{array}{l} q(t)=\\ \left\{ \begin{array}{l} \;(\varphi (t)-0.25)/20000\;\varphi (t)<-0.75\\ \sqrt{-3.98\times 10^{8}\varphi (t)+10^{8}}-10000\varphi (t)\geq -0.75and\varphi (t)<0.25\\ \;(\varphi (t)-0.25)/100\;\varphi (t)\geq 0.25 \end{array}\right. \end{array}$$

The flux-controlled memristor (Wang et al., 2012b):


(11) $$\begin{array}{l} M(\varphi (t))=\\ \left\{ \begin{array}{l} \;20000\;\varphi (t)<-0.75\\ \sqrt{-3.98\times 10^{8}\varphi (t)+10^{8}}\varphi (t)\geq -0.75and\varphi (t)<0.25\\ \;100\;\varphi (t))\geq 0.25 \end{array}\right. \end{array}$$

Substituting (10) into (9), we get (*M*=$\sqrt{-3.98\times 10^{8}\varphi \left(t\right)+10^{8}}$):


(12) $$\begin{array}{l} \tau_{M}(t)=\\ \left\{ \begin{array}{l} \;(\varphi (t)-0.25)/V_{M}\;\varphi (t)<-0.75\\ (-3.98\times 10^{8}\varphi (t)+10^{8}-10000M)/(-1.99\times 10^{8}V_{M}\;\varphi (t)\geq -0.75and\varphi (t)<0.25\\ \;(\varphi (t)-0.25)/V_{M}\;\varphi (t))\geq 0.25 \end{array}\right. \end{array}$$

**Figure 3:**
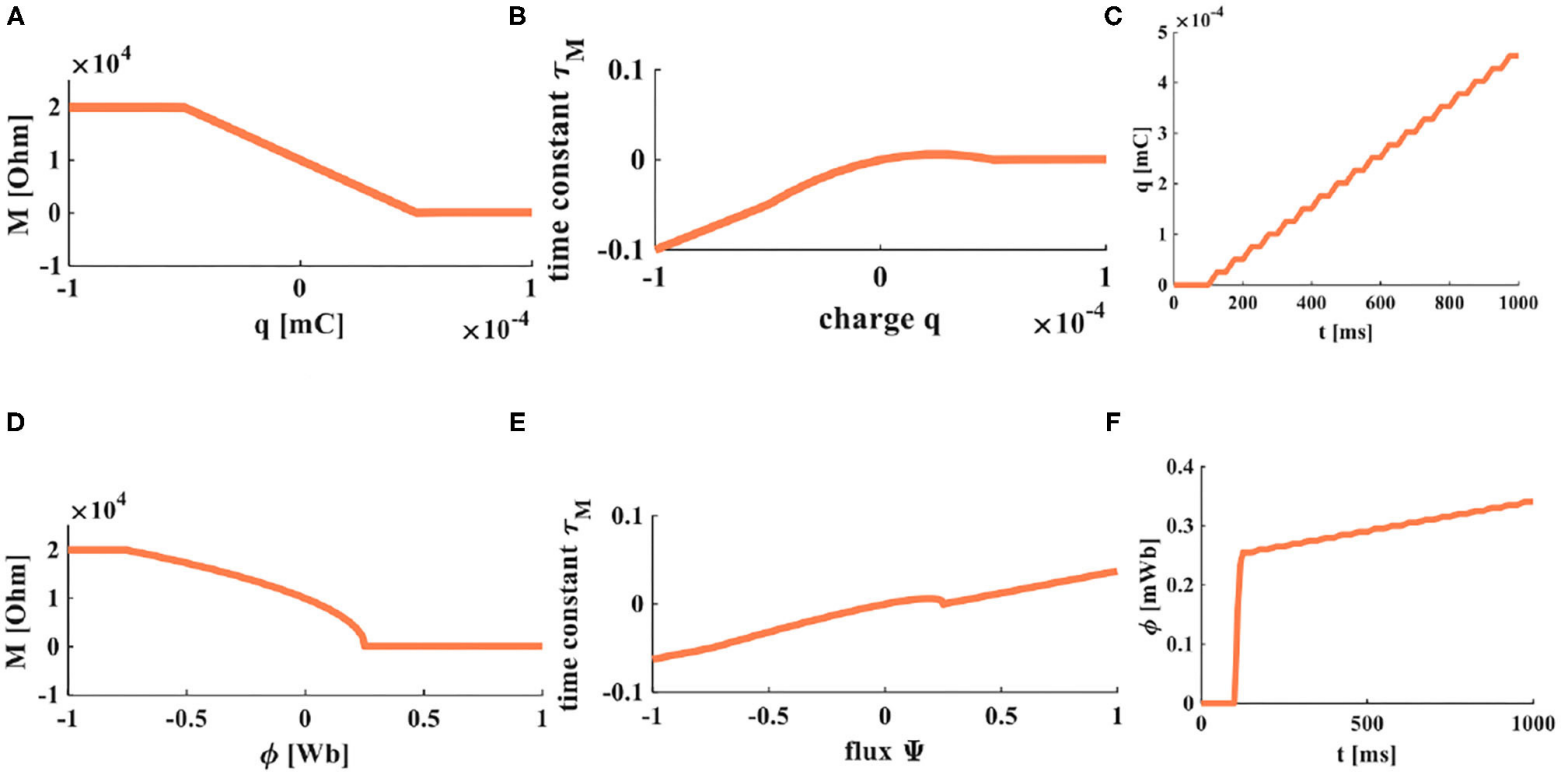
The accumulation processes of charge and flux. **(A)** The relationship between charge and memristor. **(B)** The relationship between the time constant and charge. **(C)** When a series of pulses is applied on a memristor, the accumulation process of charges. **(D)** The relationship between flux and memristor. **(E)** The relationship between the time constant and flux. **(F)** When a series of pulses is applied on a memristor, the accumulation process of flux.

From the above equations, we can get the time constants of charge-controlled and flux-controlled memristors. Their plots are shown in Figure 3.

According to the MLIF membrane circuit and (7), the mathematic expression of the MLIF model can be rewritten as follows:


(13) $$\begin{array}{r} V_{M}\left(t+\Delta t\right)-V_{M}\left(t\right)=\Delta t/\left(\tau_{M}\left(t\right)\right)\left(-V_{M}\left(t\right)+V_{rest}+M\left(t\right)I_{ext}\right) \end{array}$$

In the following experiments, the different stimuli are applied to the MLIF model, and the values of parameters will be set as *C* = 2 ×10^−9^*F*, *R* = 10^6^Ω, *V*_*rest*_ = −60*mV*, *V*_*th*_ = −50*mV*, *V*_*reset*_ = −80*mV*.

## 4. The Response of Membrane Potential to the Different External Stimuli

To compare and analyze the differential firing behaviors between the LIF and MLIF neuron models, we choose a series of pulses, the step current, a single pulse, the ramp current, and the random noise as the external stimuli (the coral red curves are external stimuli, the blue curves are membrane potentials).

### 4.1. The External Stimulus Is *I*_*ext*_ = 0*nA*

The initial membrane potentials are −80 and −50 mv and are applied to the MLIF model.

**Figure 4:**
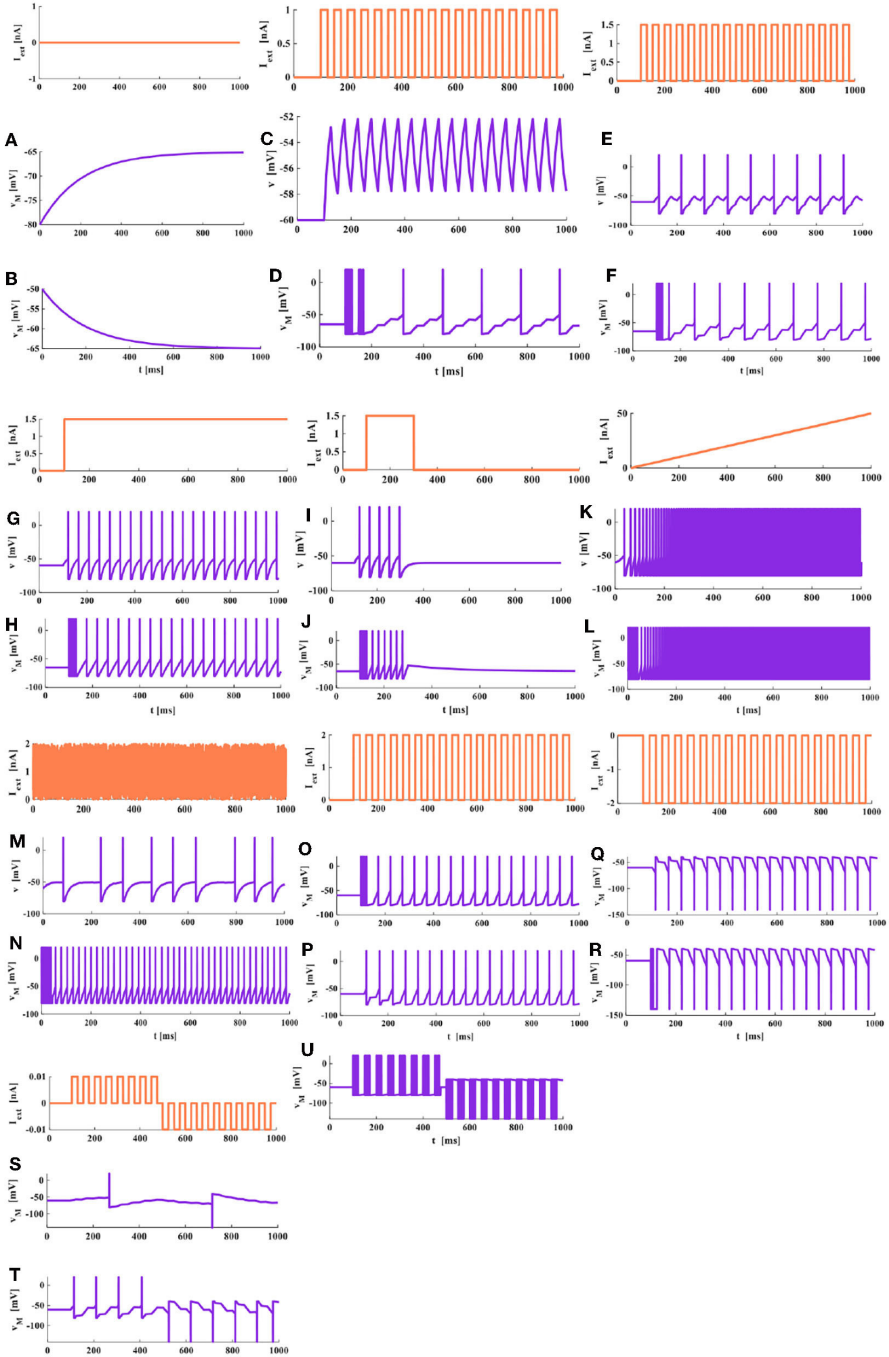
The relationship between the external stimulus and the action potential (the threshold voltage is −50 mV, the peak value of the action potential is 20 mV, and the reset potential is −80 mV). **(A)** The initial potential is −80 mV. **(B)** The initial potential is −50 mV. **(C)** A series of pulses act on the LIF spiking model. **(D)** A series of pulse act on the MLIF spiking model. **(E)** A series of pulses are injected into the LIF spiking model. **(F)** A series of pulses are injected into the MLIF spiking model. **(G)** The step current is applied to the LIF spiking model. **(H)** The step current is applied to the MLIF spiking model. **(I)** The LIF spiking model receives a single pulse. **(J)** The MLIF spiking model receives a single pulse. **(K)** The ramp stimulus acts on the LIF spiking model. **(L)** The ramp stimulus acts on the MLIF spiking model. **(M)** The random noise is applied to the LIF spiking model. **(N)** The random noise is applied to the MLIF spiking model. **(O)** Forward input pulse and forward memristor. **(P)** Forward input pulse and reverse memristor. **(Q)** Reverse input pulse and forward memristor. **(R)** Reverse input pulse and reverse memristor. **(S)** The external stimulus is 0.01 nA acts on the MLIF spiking model. **(T)** The external stimulus is 0.045 nA. **(U)** The external stimulus increases to 2 nA.

When the neuron cell membrane is at the resting state, it has a strong negative potential inside, and its value is about −65*mV*, which is called the resting potential. When the membrane potential value decreases in the negative direction, it is called depolarization of the cell membrane. The membrane potential changes from −80 to −65*mV* (Figure 4A). When the membrane potential value increases negatively, it is called hyperpolarization of the cell membrane. The membrane potential varies from −50 to −65*mV* (Figure 4B). According to the simulation results, we can conclude that no matter what the initial value is, the membrane potential always returns to the resting state in the end. The speed at which the membrane potential returns to the resting potential depends on the time constant τ_*M*_, and the larger value of τ_*M*_, the longer time consumed.

### 4.2. The External Stimulus Is a Series of Pulses: *I*_*ext*_ = 1*nA*

Under the same values of parameters, we compare the LIF model and the MLIF model.

In Figure 4C, the LIF spiking model has no action potential generated, which means the current with a small amplitude applied to the LIF model cannot promote the membrane potential to reach the threshold. When the external stimulus current is applied to the MLIF spiking model, it can generate action potentials in Figure 4D. When the first two pulses arrive, a series of dense action potentials are produced, and it has four or five action potentials in each cluster. This phenomenon is called biological spiking frequency adaptation in a neuron. After that, it fires continuously relative stable action potentials with the identical waveform.

### 4.3. Increase the Amplitude of External Stimulus Current, a Series of Pulses: *I*_*ext*_ = 1.5*nA*

When the strength of the external current is increased from 1 to 1.5 nA, the action potentials of both models are produced at regular intervals. No biological spiking frequency adaptation is generated in the LIF model (Figure 4E). The LIF model has 9 individual spikes in 1,000 ms. When the first pulse arrives, the dense spikes with five spikes, about 25 ms adaptation process are produced in the MLIF model (Figure 4F). After that, the single spike is generated regularly. Therefore, the MLIF model has good biological frequency adaptation. We can consider a realistic biological situation. The firing spikes are activated by the previous spikes (the generation of the membrane potential is caused by the linear superposition of the presynaptic spikes). Once the action potential is initiated, it is propagated from one neuron to another. Therefore, the generation of every action potential is the result of multiple presynaptic pulses.

### 4.4. The External Stimulus Is Step Current: *I*_*ext*_ = 1.5*nA*

When the external stimulus is the step current, the two models can produce the action potentials. The mean firing rate of the MLIF model (Figure 4H) is higher than that of the LIF model (Figure 4G). The MLIF model has a good adaptation process before regularly fires, similar to the biological neuron self-firing property. The typical LIF model cannot capture this adaptation process (Connors and Gutnick, 1990).

### 4.5. The External Stimulus Is a Single Pulse Input: *I*_*ext*_ = 1.5*nA*

When the LIF neuron model (Figure 4I) is compared with the MLIF neuron model (Figure 4J), they can produce the action potentials. The MLIF model performs a high firing frequency and shows good biological frequency adaptation, but the LIF model has no adaptation process.

### 4.6. The External Stimulus Is a Ramp Stimulus

The ramp stimulus is applied to the LIF model and the MLIF model (Figures 4K,L). With the increase of the external stimulus, the action potentials of the two models become dense, and the number of spikes increases quickly. The MLIF spiking model generates 7 action potentials from 0 to 45 ms; it is comprehended as the biological frequency adaptation process. The MILF model has a high firing frequency and good biological frequency adaptation. The LIF model has no biological frequency adaptation.

### 4.7. The External Stimulus Is Random Noise

When the neurons receive the proper stimulus, the membrane potential reaches the threshold potential. The action potential is generated, propagating along the axon without waveform change and transmission loss to the axon terminal to activate other neurons. The biological nervous system is a kind of system with non-linear noise. To mimic the real biological phenomenon, we use stochastic noise as the input stimulus. Under the influence of noise, the LIF membrane model does not have a biological adaptation process. The intrinsic spike interval is distinct because the noise stimulus is random (Figure 4M). The MLIF membrane model generates dense action potentials between 0 and 50 ms after that generates a series of action potentials regularly (Figure 4N). The MLIF model performs a higher firing frequency and good biological frequency adaptation.

### 4.8. The External Stimulus Is a Series of Pulses: *I*_*ext*_>0 and *I*_*ext*_ < 0

We increase the strength of external stimulus to 2 nA, and other values of parameters have no change. The neuron may receive the excitatory signals or the inhibitory signals from other neurons. As long as the membrane potential exceeds the given threshold potential, it will produce the firing behaviors. In **Figures 4O,R**, the MLIF model receives the forward and reverse stimuli, and the polarity of the memristor is forward and reverse, respectively. Under these situations, the memristor will change from a high-memristance state to a low-memristance state. The initial value of memristance is large, which will lead to the high-firing frequency generated at the beginning of the waveforms. The MLIF model has better biological adaptation in both cases and shows similar waveforms, except the directions of waveforms are distinct. In Figures 4P,Q, the forward and reverse stimuli are used as injected currents, the polarity of the memristor is reverse and forward, respectively. The memristor will change from a low-memristance state to a high-memristance state. The initial value of memristance is small, and low-frequency firing is produced. No biological frequency adaptation is generated at the beginning of the waveforms.

### 4.9. The First Half of Period Is Forward Stimulus While the Second Half of Period Is Reverse Stimulus

The previous half of the external stimulus is a series of forward pulses; the latter half is reverse pulses. When the external stimulus is small (0.01 nA), the MLIF model can generate one forward action potential and one reverse action potential (Figure 4S). With the increase of the external stimulus, more action potentials are generated. Every action potential is produced by a neuron, which is stimulated by a set of presynaptic spikes, where it behaves more like a realistic neuron. When the external stimuli are 0.045 nA, the MLIF spiking model generates many spikes (Figure 4T). The current stimulus becomes 2 nA, the MLIF model generates high-frequency repetitive bursts with four or five spikes (Figure 4U). It is similar to the spiking pattern generated by the fast rhythmic bursting neuron. Various spiking patterns are reproduced with distinct stimulus intensities.

## 5. The MLIF Cable Model for the Propagation of Electrical Signals

The emergence of the cable theory is due to the derivation and application of the cable formula, originally designed to solve the first transatlantic telegraph cable calculations. In the 1930s, Cole, Rushton, and Hodgkin, and other scholars provided vital experimental evidence for the relevance between cable theory and nerve axons (Koch, 1998; Tasaki and Matsumoto, 2002). It is an essential reference for studying excitable neurons and helps us understand the role of excitability (Koch, 1998).

**Figure 5:**
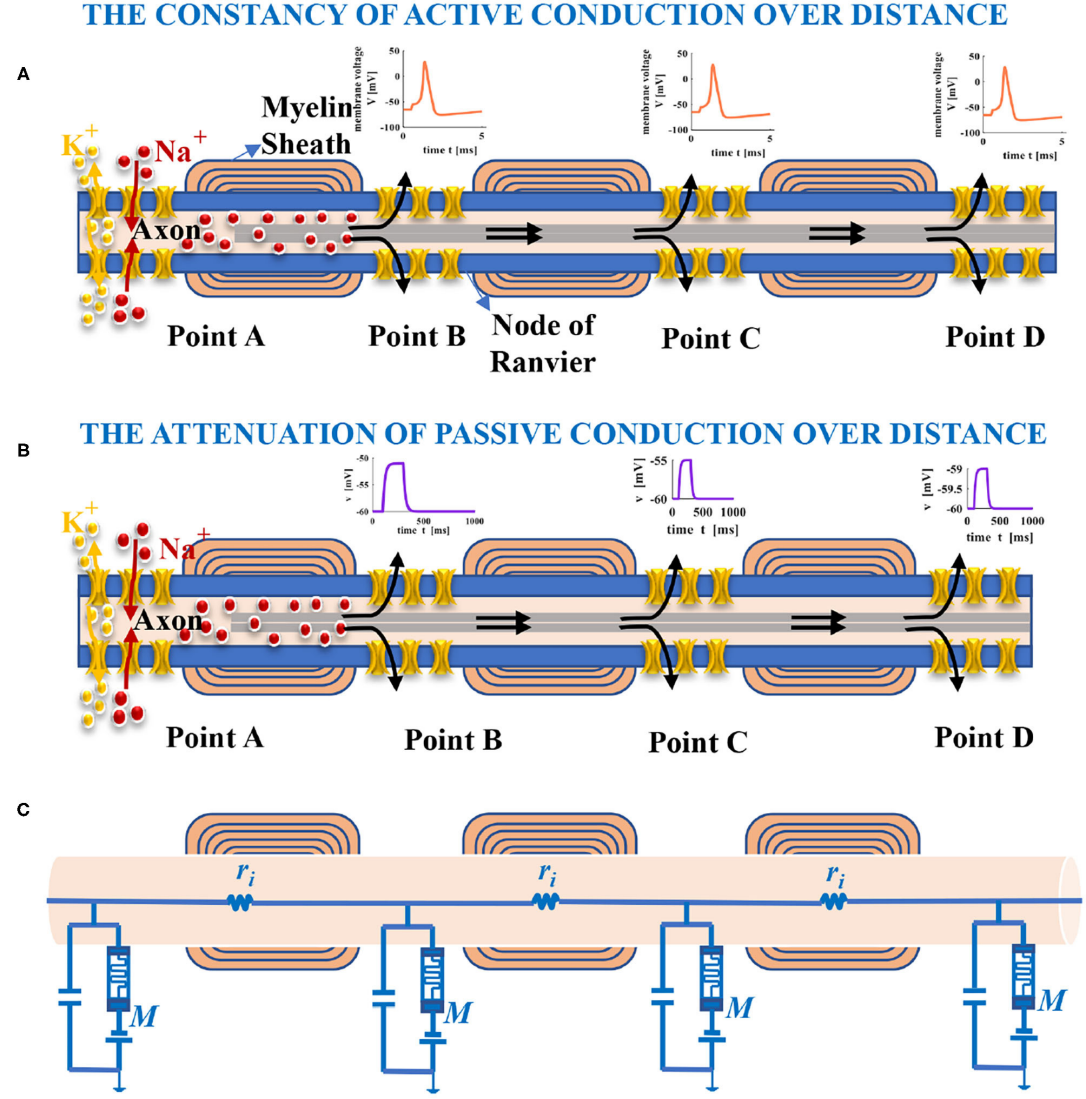
Long-distance propagation of spikes in the axon. **(A)** The action potential with constant amplitude yielded by the external current pulse, which spreads with increasing distance (the black arrows show the conduction direction of current). **(B)** The amplitude of membrane potential decreases with the distance increase because the injected current leaks out of the axon. **(C)** An electrical circuit model of the axon in propagation.

Most neurons have only one axon, which can be up to one meter in length and covered by myelin sheath partially. The part without myelin sheath is called the node of Ranvier (namely, point A, point B, point C, and point D), as shown in Figure 5A. Spikes are generated at the start of the axon shaped like a small bump and propagate along the axon. The passive and active electrical properties in a neuron are determined by the voltage change resulting from the external current pulse passing across the axonal membrane. If the external current pulse is large enough to stimulate an action potential, the action potential with constant magnitude will propagate along the entire axon (Figure 5A) (Purves, 2018). We suppose the external current pulse is too small to evoke an action potential. In that case, the amplitude of the resulting potential will attenuate with increasing distance from the site of current injection (Figure 5B) (Purves, 2018). The neuron can be modeled as a cable. For the part of the axon, we can think of it as many cylindrical compartments coupled to each other with resistors (Figure 5C) (Purves, 2018). The node of Ranvier is modeled as the MLIF model. The interior of the axon is filled with axoplasm, which is the internal pathway for ions to flow along. It can be represented as an electrical resistor. The primary function of the axial resistor is to distinguish the interior and the exterior of the model and influence the waveform of membrane potentials.

Nodes of Ranvier use the mechanism of saltatory conduction to speed up the propagation of action potentials. It is also the vulnerable site of the axon and is prone to cause neurological injury for lacking myelin protection (Lubetzki et al., 2020).

Myelin sheaths serve as the electrical insulator between adjacent axons to avoid interference and further enhance the saltatory conduction (Chang et al., 2016) and protect the axon or surrounding tissues.

The non-linear memristor with a tunable resistance performs good synaptic plasticity in corresponding to continuously modulate the synaptic weight of biological synapses (Kim et al., 2017); it can be used to mimic the artificial synapses (Jo et al., 2010; Wang et al., 2012a; Cai and Tetzlaff, 2014; Yang et al., 2017; Deswal and Kumar, 2019) and realize the single-component synapse emulators (Serb et al., 2016). In neuroscience, synaptic plasticity refers to a change in the strength of the connection between two neurons. STDP is a vital learning rule for regulating and controlling synaptic weight (Guyonneau et al., 2005). A memristor is a device with synaptic characteristics whose continuously variable conductance can be used to simulate synaptic weight and realize the STDP learning rule (Wang et al., 2014) acquired in biology (Bi and Poo, 2001). A memristor performs the property of the STDP learning rule, the voltage threshold *V*_*th*_ = 1 V). Thereby it can be used as a synapse between neurons.

**Figure 6:**
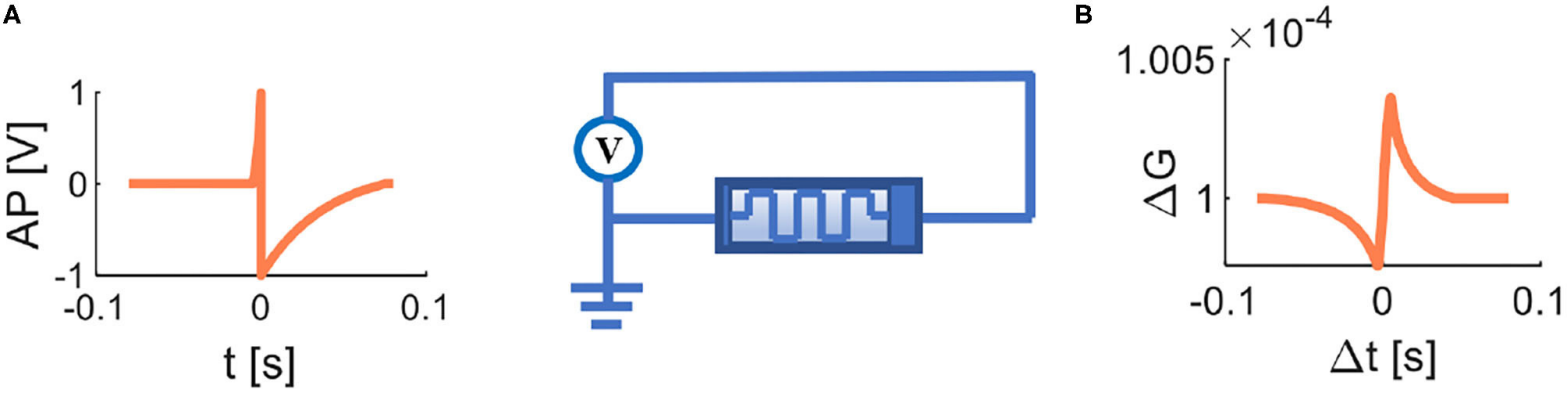
The memristive STDP. **(A)** The voltage is applied to the memristor. **(B)** STDP learning curve of a memristor.

When the action potential (Figure 6A) acts on the memristor, and the STDP learning curve (Figure 6B) of a memristor is realized. Here, AP means the action potential, Δt is the time difference between a postsynaptic spike and a presynaptic spike, and ΔG denotes the memductance. When Δt > 0 (the presynaptic potential takes precedence over the postsynaptic potential), ΔG increases with the decrease of Δt. When Δt < 0 (the presynaptic potential lags behind the postsynaptic potential), ΔG decreases with the increase of Δt. The obtained STDP learning plot is similar to the curve measured biologically, and the memristor can implement the STDP learning rule. All of the parameter values and formulas in simulation refer to Wang et al. (2014).

**Figure 7:**
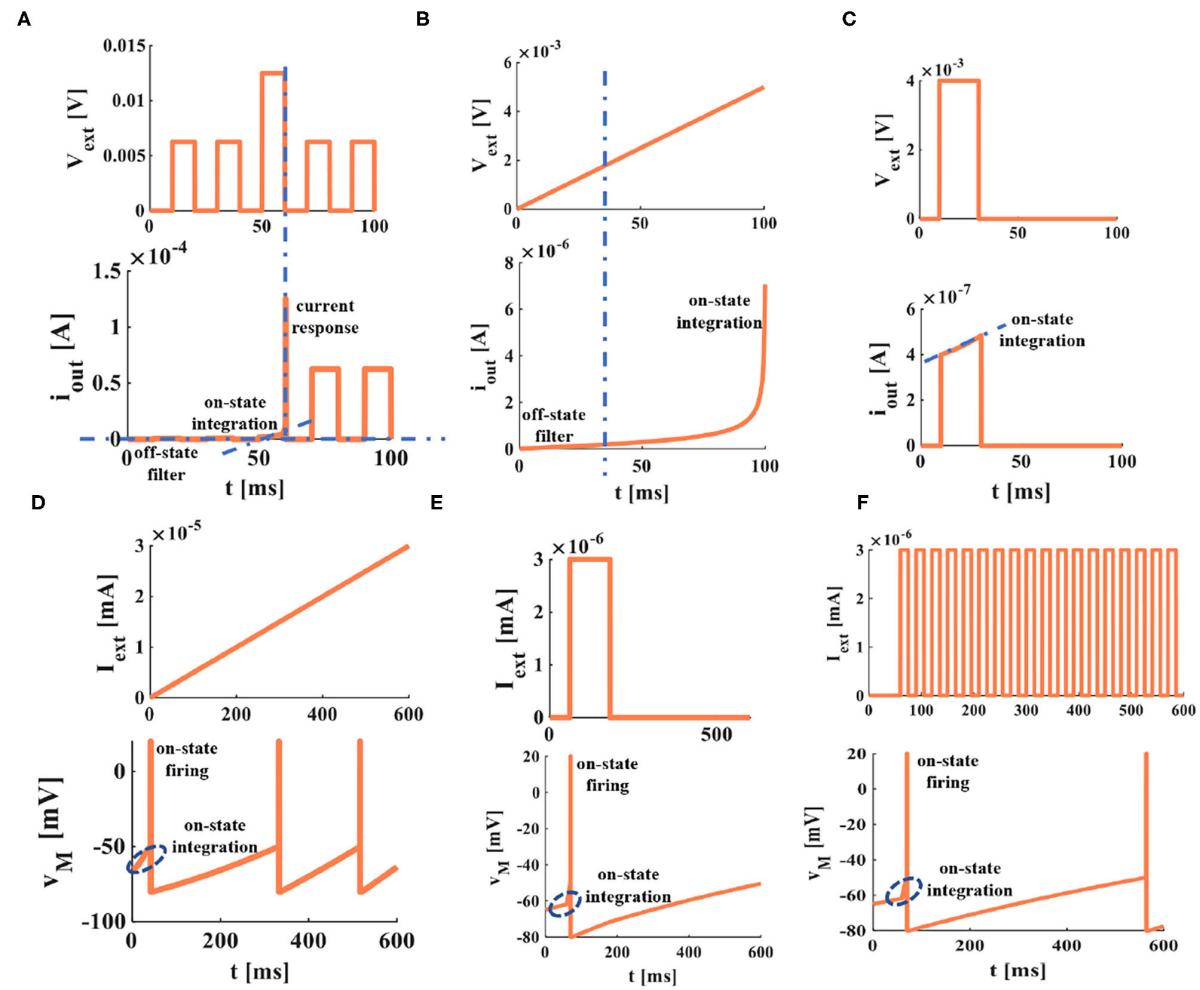
Memristive dendrite and memristive soma. **(A)** The non-linear filtering and integration of dendrite. **(B)** The non-linear integration of dendrite. **(C)** The current response of memristive dendrite in on and off states. **(D)** The voltage response of a ramp current applied on the memristive soma. **(E)** The voltage response of a single current pulse applied to the memristive soma. **(F)** The voltage response of a series of current pulses applied to the memristive soma [each plot in this figure is divided into the figure above (the external stimulus applied to the memristor) and the figure below (the response of a memristor to the external stimulus)].

**Figure 8:**
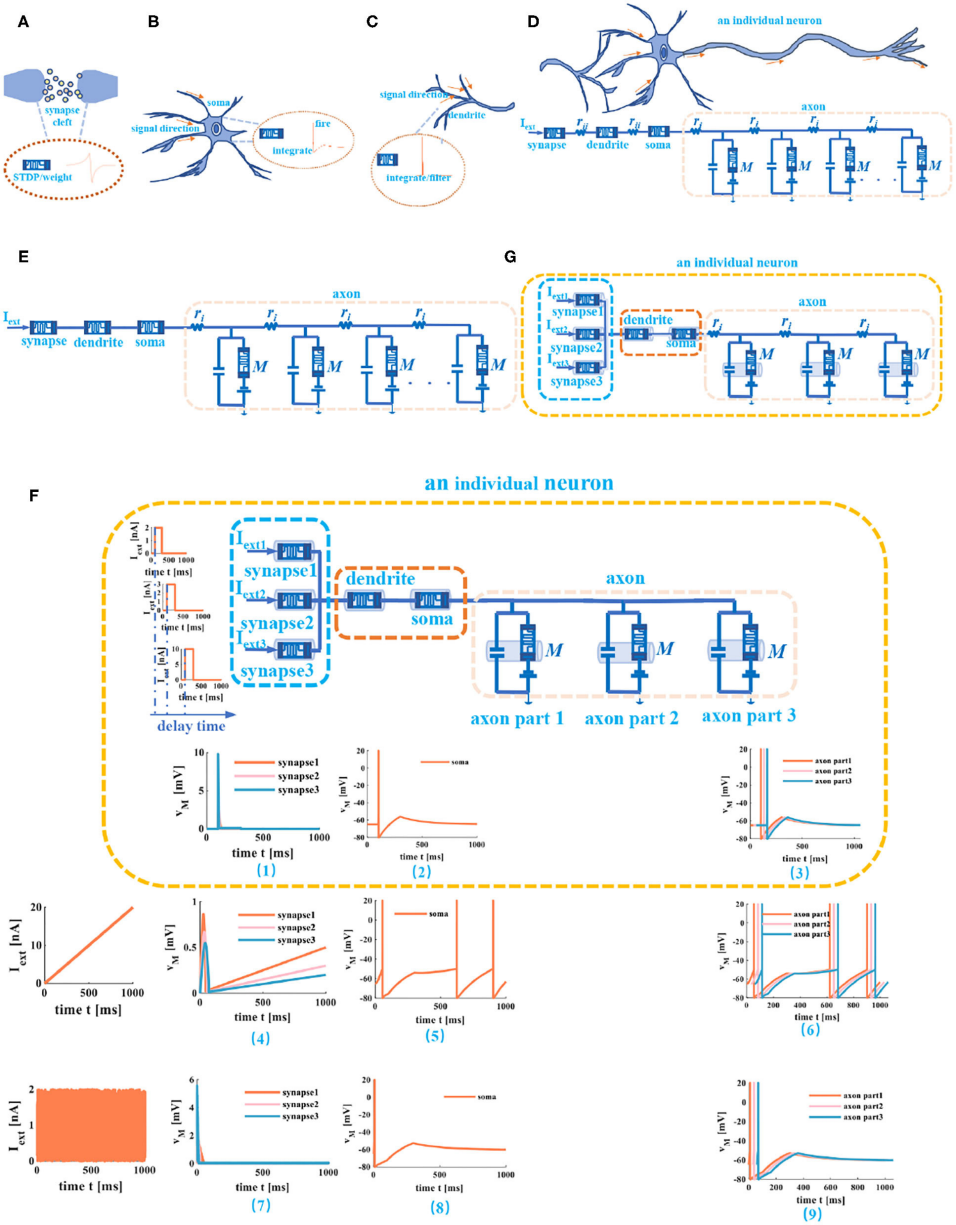
Memristive cable model of a neuron. **(A)** The memristor used as the synapse. **(B)** The memristor is used as soma. **(C)** The memristor used as dendrite. **(D)** An individual neuron realized by memristive synapse, memristive dendrite, memristive soma, and memristive axon. **(E)** An individual neuron without the humoral resistance and the cytoplasmic resistance. **(F)** The three different current stimuli act on the compartmental circuit model of a neuron. **(G)** An individual neuron circuit model with three synapses.

In Li et al. (2020), an artificial dendrite is implemented by a memristor with a *Pt*/*TaO*_*x*_/*AlO*_δ_/*Al* structure, an artificial soma is fabricated with a Mott memristor with a NbOx structure, and an artificial synapse realized by a memristor with a *HfO*_*x*_ structure. A fully memristive spiking neuron structure is designed only by drift and diffusion memristors without any other electron elements (Tang et al., 2019). It means that an individual memristor can completely mimic the synapse, dendrite, and cell body functions in the propagation of electrical signals. Here, the non-volatile memristors (Wang et al., 2012b) are used as the dendrite and the soma (Figures 8B,C), the simulation results perform the electrical properties of artificial dendrite and soma. In Figures 7A–C, a series of voltage pulses, the ramp voltage, and a single voltage pulse are applied to the memristive dendrite, respectively. The memristive dendrite receives a series of voltage pulses. When the voltage pulses are small (0.00625*V*), the dendrite device is in the off-state, no current response is activated, the memristor is an off-state filter. Until a large voltage pulse (0.0125*V*) arrives, the memristor is in the on-state, giving rise to the integral behavior (the position of the blue oblique dashed line), and the current response with a larger amplitude is produced (the vertical blue dotted line in Figure 7A). After that, the current responses of small voltage pulses (0.00625*V*) can be observed (Figure 7A). In Figure 7B, when the slope voltage is low, the memristor is turned off and shows an apparent filtering phenomenon in the time duration from 0ms to 21*ms* (the position of the blue dotted line in Figure 7B). After that, the memristor is turned on; the integration phenomenon can be observed (the plot shows an upward trend). A single pulse voltage (0.004*V*) acts on the memristive dendrite. The memristor is on-state and performs the property of non-linear integration (the position of the blue dotted line), as shown in Figure 7C. These simulations show the properties of non-linear integration and filtering of the memristive dendrite.

Comparing the experimental results in Figure 7 with those in Li et al. (2020), they agree with each other. It verifies that the non-linear integration and the filtering function of a memristive dendrite, that is, a single memristor can act as an artificial dendrite.

The differences between the memristive dendrite and artificial dendrite device in Li et al. (2020) are:

1. The author of Li et al. (2020) developed a memristor with a new material structure to fabricate a dendrite device; we use the original flux-controlled memristor to implement the memristive dendrite.
2. The external voltage applied to the artificial dendrite in Li et al. (2020) is from 2*V* to 5*V*; the external voltage we use is two or three orders of magnitude smaller.

The soma is the functional part of integration and firing (Li et al., 2020) in a neuron. To mimic the soma of a biological neuron, we set the voltage threshold as −50*mV*, the firing spike potential as 20*mV*, the external stimulus as 3*nA*, the resting potential as −65*mV*, and the time duration as 0*ms* to 600*ms*. The simulation plots in Figures 7D–F perform that the voltage responses to the different current stimuli. When the external stimulus is large enough, the memristive soma can activate the action potential. A short upstroke waveform before firing (inside the blue ellipse with a dotted line) shows the non-linear integration of the memristive soma. Therefore, a memristor can mimic the firing and integration functions of the soma.

The computational model of a neuron is a vital tool to unveil the mysteries of the human brain and a crucial way to extend to any field beyond neuroscience (Poirazi and Papoutsi, 2020). Meanwhile, many theoretical studies have confirmed that an individual neuron can act as a powerful computing unit (Murphy and Miller, 2003; Duan et al., 2020).

Any neuronal structure that contains the dendrite, the soma, and the axon can be described as a compartmental cylinder model (Tuckwell, 1988). By connecting these cylinder models, the cable model is implemented (Figure 8D). Here, the memristor acts as the synapse (Figure 8A), the soma (Figure 8B), and the dendrite (Figure 8C). At the same time, ignoring humoral resistance (*r*_*ii*_) between synapse and dendrite, the cytoplasmic resistance (*r*_*ii*_) between dendrite and soma (because the coupling conductance is too small to generate spikes) (Liu and Tian, 1996). The axon is modeled by the “T” circuits (the MLIF unit and two axial resistors; Figure 8D).

Here, we do not consider the complex phenomena of the axon, such as the failure of action potential propagation, the reflection of action potentials, and the variability of axonal morphology (Dominique, 2004). The axon is regarded as the stable transmission cable of the action potential once the action potential is evoked. The cable model consists of a series of short compartmental cylinders. We take the active propagation of a neuron as an example and apply three distinct external stimuli through synapses to a neuron.

(1) We ignore the axial resistance ri in Figure 8E, the axon consists of three MLIF spiking models, and the simple MLIF cable model is obtained (Figure 8F). The three single pulses (from small to large: 2, 3, and 10*nA*) with a 30*ms* delay are applied to the three memristive synapses. The voltage responses of memristive synapses are obtained [the plot (1) at the bottom of the neuron circuit model in Figure 8F]. The greater the external stimulus, the greater the voltage response. After that, the total current passes through the dendritic and somatic compartments, and the action potential is generated [plot (2) at the bottom of the neuron circuit model in Figure 8F]. Finally, the action potential propagates along the axon, and the action potentials without any distortion are obtained through the MLIF axonal compartments [axon part 1, axon part 2, and axon part 3 of the plot (3) in Figure 8F]. When the ramp input (the peak value varies from small to large: 2, 3, and 5*nA*) and the noise input (the amplitude value goes from small to large: 2, 4, and 6*nA*) with a 30-ms delay act on the memristive neuron, the voltage responses of synapses and the action potentials are achieved [plots (4), (5), (7), and (8) in Figure 8F]. The MLIF spiking models realize the signal transmission of the axon [the plot (6) and the plot (9) in Figure 8F]. These simulations prove that the MLIF cable model of the neuron is successful and can completely mimic the information transmission in a neuron.

(2) The axial resistance ri in Figure 8E is considered (Figure 8G). According to the cable theory, the cable equation in the active state can be expressed as follows (Matsumoto and Tasaki, 1977; Dayan and Abbott, 2001):


(14) $$\begin{array}{r} C_{m}\partial v/\partial t=1/r_{i}\partial^{2}v/\partial x^{2}-v/R_{m}+i_{ext} \end{array}$$

*C*_*m*_ is the membrane capacitance, *R*_*m*_ is the resistor of the LIF spiking model, and iext is the external stimulus.

Replacing *R*_*m*_ with *M*, (14) can be rewritten as:


(15) $$\begin{array}{r} C_{m}\partial v/\partial t=1/r_{i}\partial^{2}v/\partial x^{2}-v/M+i_{ext} \end{array}$$

And then, multiply (15) by *M*


(16) $$\begin{array}{r} MC_{m}\partial v/\partial t=M/r_{i}\partial^{2}v/\partial x^{2}-v+Mi_{ext} \end{array}$$

The *M* is the memristor of the MLIF spiking model, the membrane time constant τ_*m*_ = *MC*_*m*_, the membrane space constant λ=$\sqrt{M/r_{i}}$, *v* = *V*_*M*_ - *V*_*rest*_. *V*_*M*_ is the membrane potential, and *V*_*rest*_ is the resting potential.

To simpLIFy (16), we assume the cable is infinite. The external current is injected, the solution of the equation is independent of time, and the cable equation can be rewritten as follows (Liu and Tian, 1996; Dayan and Abbott, 2001):


(17) $$\begin{array}{r} M/r_{i}\partial 2v/\partial x^{2}=v-Mi_{ext} \end{array}$$

We solve the second-order differential Equation (17) and get the general solution $V_{1}e^{-x/\lambda }$ + $V_{2}e^{x/\lambda }$. For the region *x*>0, which means that *V*_2_ = 0. For the region *x* < 0, which means that *V*_1_ = 0. At *x* = 0, which denotes the site of current injection. To obtain the continuous solution, it must satisfy *V*_1_ = *V*_2_ = *V*_0_, then the expression $v\left(x\right)=V_{0}e^{-|x|/\lambda }$. The special solution is *Mi*_*ext*_. the solution of (17) is $v\left(x\right)=V_{0}e^{-|x|/\lambda }+Mi_{ext}$. here, we only consider the situation of *x*>0, so $v\left(x\right)=V_{0}e^{-x/\lambda }$.


(18) $$\begin{array}{r} v\left(x\right)=V_{0}e^{-x/\lambda }=ire^{-x/\lambda }=i{\left(Mr_{i}\right)}^{1/2}e^{-x/\lambda } \end{array}$$

The *v* is the electrotonic potential, *i* is the injected current, *r* is the input resistance of nerve fiber, *M* and *r*_*i*_ contribute to the input memristance, *x* is the distance from the current source, and λ is the length constant of the fiber (the parameter values are listed in Table 1).

**Table 1:** The parameters of the MLIF cable axon.

**Parameters**	**Experimental values**	**Descriptions**
*C*_*m*_	1μ F	Membrane capacitance
M	*M*_*max*_ = 20, 000*Ohm* *M*_*min*_ = 100*Ohm*	Membrane resistance
*r*_*i*_	100 Ohm	Axoplasm resistance
*V*_*rest*_	−65 mV	Resting membrane potential
*V*_*th*_	−50 mV	Firing threshold potential
*V*_*spike*_	20 mV	Peak value of the membrane potential

To observe the firing and propagation behaviors of electrical signals in a cable axon, we select a series of pulses, the rump current, and the noisy input to act on a cable neuron. The current stimulus is large enough to stimulate the action potentials, and the action potentials propagate steadily along the axon with the constant amplitude and waveform.

**Figure 9:**
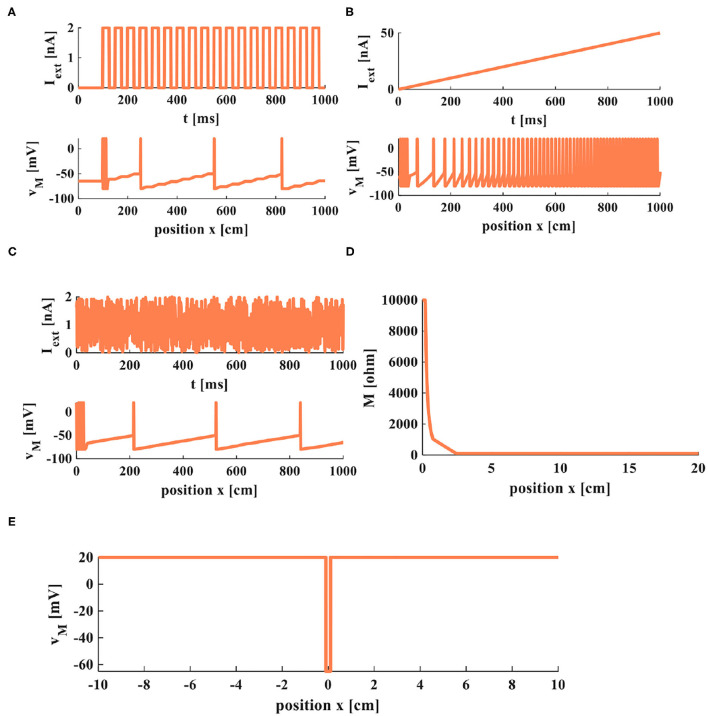
Firing and propagation of electrical signals in a cable axon. **(A)** A series of pulses applied on the cable axon. **(B)** The rump current is applied to the cable axon. **(C)** The noisy input is applied to the cable axon. **(D)** The relationship between memristor and the position. **(E)** The relationship between membrane potential and the position.

The action potentials are evoked by the external stimuli (Figures 9A–C). The value of memristor changes from 10, 000*Ohm* (the initial value) to 100*Ohm* with the distance increasing (Figure 9D). The amplitude of the action potential keeps constant at distinct positions, as shown in Figure 9E. At the point *x* = 0, the membrane potential is at the resting state, *v*(0) = −65*mV*. The current pulses extend in both directions from the injection point of current (*x* = 0) in the nerve fiber. The amplitude of the response potential maintains constant as the distance increases, *v*(*x*) = 20*mV*.

The simulation results indicate that the MLIF cable model can efficiently imitate the propagation of electrical signals and the firing process of neurons.

## 6. The Generation and Recognition of the Morse Code for the Alphabets in the MLIF Model

**Figure 10:**
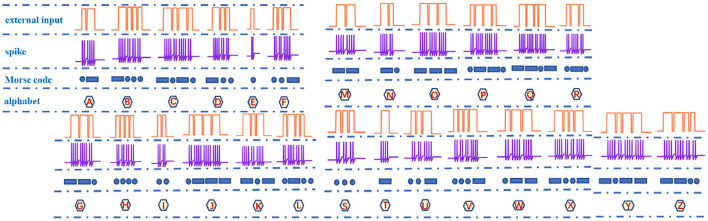
The correspondence relationship of letters, spikes, and Morse codes.

The information in the brain is encoded into pulse sequences, which are equivalent to the Morse code (Cles, 1992). Therefore, we utilize the action potential (a series of spike sequences) generated by the MLIF spiking model (Figure 2A, the resting potential −65*mV*, the threshold voltage −50*mV*, and the reset potential −80*mV*) to realize the representation of Morse code. When the external pulse intensity is constant (13*nA*), the different pulse widths can produce specific numbers of action potentials. The Morse code consists of the dot (.) and dash (-) markers. We select the four-spike group represents the dash and the two-spike group denotes the dot. The four-spike group and the two-spike group in action potentials are caused by applying the long-time (0.1*ms*) current pulse and the short-time (0.05*ms*) current pulse (Hasdak et al., 2018; Tan et al., 2020) (Figure 10).

Spike counting is an effective way to realize the Morse code, which is extensively used in biology (Tan et al., 2020). We take the letter “A” as an example, and its Morse code consists of a dot and a dash. The short current pulse and the long current pulse (two coral pulses in Figure 10) act on the MLIF spiking model, and the two voltage pulses and four voltage pulses are evoked (the blue firing spikes in Figure 10). The two-spike action potential corresponds to a dot, and the four-spike action potential corresponds to a dash in Morse code (the dark blue geometric patterns in Figure 10: a circle and a rectangle). Then the Morse code of the letter “A” can be represented by the action potentials. Finally, the total number of spikes describes each letter and can be applied to interpret the action potentials into the Morse code. 26 English letters are implemented in Figure 10.

**Figure 11:**
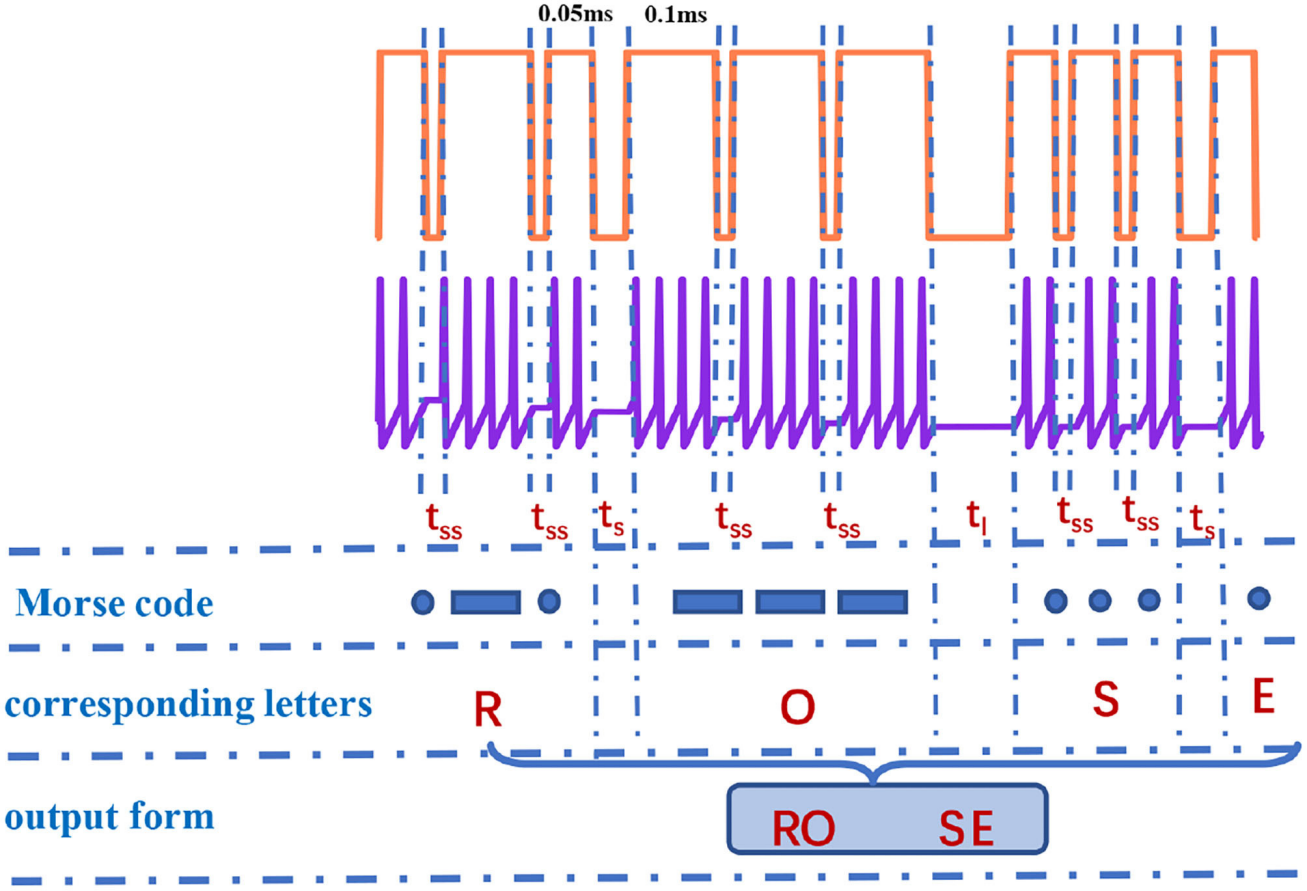
The recognition of “RO” and “SE” in Morse code [the action time of a series of pulses is 1,000 ms (the coral red plot)].

It is observed that the total number of spikes in every letter is not distinct in Figure 10. The total number of spikes in letters “D”, “F”, “H”, “M”, “R”, and “U” is 8, the total number of spikes in letters “B”, “G”, “K”, “L”, “V”, and “W” is 10, the total number of spikes in letters “C”, “O”, “P”, “X”, and “Z” is 12, the total number of spikes in letters “I” and “T” is 4, the total number of spikes in letters “J”, “Q”, and “Y” is 14, and the total number of spikes in letters “A”, “N”, and “S” is 6. The internal-spike interval is the length of time between the generated action potentials. It can be used to identify whether the produced action potential is a single letter or a word. Therefore, we need to consider the pulse time intervals in the simulation (*t*_*ss*_, *t*_*l*_, and *t*_*s*_) to recognize the Morse code correctly. The *t*_*ss*_ means the internal time interval of the Morse code. The long- and short-time intervals (*t*_*l*_, *t*_*s*_) without spikes indicate the spacing between words and between letters, accordingly (Figure 11). In our simulation, we assume that “RO” and “SE” are two words separated by the long-time interval.And the current pulse interval is 0.02*ms* (*t*_*ss*_), the width of narrow pulses is 0.05*ms*, the width of wide pulses is 0.1*ms*, the interval between letters is 0.04*ms* (*t*_*s*_), and the interval between words is 0.1*ms* (*t*_*l*_). The current pulses are applied to the MLIF spiking model, and the action potentials (two-spike and four-spike groups) are generated. The input current signal is transformed into the action potential. The Morse code can be obtained according to the corresponding relationship between two or four pulses and Morse code similar to the information coding process. We take the “R” as an example, and the three continuous current pulses (narrow-wide-narrow pulse) are applied to the MLIF neuron model, the action potentials are achieved (two-spike, four-spike, and two-spike groups). Two-spike group corresponds to the dot, and the four-spike group corresponds to the dash. The Morse code of “R” is denoted the dot-*t*_*ss*_-dash-*t*_*ss*_-dot.

The obtained Morse code can be interpreted as letters based on the relationship between Morse code and letters and the intervals between letters and words. We take the “RO” and “SE” as an example, and they consist of dots and dashes. When the time interval is *t*_*ss*_ between dots and dashes, it means these dots and dashes form a letter (such as “R”, dot-dash-dot). The time interval *t*_*s*_ appears in dots and dashes, it denotes two letters (such as “RO”, dot-dash-dot-*t*_*s*_-dash-dash-dash). The time interval *t*_*l*_ occurs between dots and dashes, it represents two words (such as “RO” and “SE”, dot-dash-dot-*t*_*s*_-dash-dash-dash-*t*_*l*_-dot-dot-dot-*t*_*s*_-dot). We can recognize the Morse code effectively. Finally, the generation and recognition of Morse code can be implemented effectively.

## 7. Conclusion

In this work, the LIF model with the non-volatile memristor is proposed successfully, and we aim to develop the application of memristor in neuroscience. We choose the flux-controlled memristor to combine with the LIF spiking model and get the MLIF spiking model. To demonstrate the superiority of the MLIF model over the LIF model, we compared the firing patterns of the two models. The simulation results show that the MLIF model has good biological spiking frequency adaptation, higher firing frequency, and rich firing patterns. The MLIF model can reproduce the firing behavior of biological neurons very well.

Due to the intrinsic characteristics of a memristor, it can potentially promote the analysis and application of biological neural models. This work has experimentally proved that a single memristor can be used as a synapse, performs the function of integration and filtering of the dendrite, and realizes the function of integration and firing of soma. An individual neuron constructed entirely by memristors can emulate passive and active propagations over time; finally, it efficiently transmits the information. Our MLIF model converts the current pulses to potential spikes, corresponds to the Morse code sequence. The simulation results indicate that the MLIF model can successfully generate and recognize the Morse code. Therefore, the proposed MLIF model can be a potential building block for reproducing the behaviors of a biological neuron and constructing the spiking neural networks.

## Data Availability Statement

The original contributions presented in the study are included in the article/supplementary material, further inquiries can be directed to the corresponding author/s.

## Author Contributions

XF built models and simulations, carried out the experimental analysis, and prepared the manuscript in this work. DL, SD, and LW supervised the content of the article and the results of the simulations. All authors contributed to the article and approved the submitted version.

## Funding

Project supported by the National Key R and D Program of China (Grant No. 2018YFB1306600), the National Natural Science Foundation of China (Grant Nos. 62076207, 62076208, and U20A20227), and the Fundamental Science and Advanced Technology Research Foundation of Chongqing, China (Grant No. cstc2017jcyjBX0050).

## Conflict of Interest

The authors declare that the research was conducted in the absence of any commercial or financial relationships that could be construed as a potential conflict of interest.

## Publisher's Note

All claims expressed in this article are solely those of the authors and do not necessarily represent those of their affiliated organizations, or those of the publisher, the editors and the reviewers. Any product that may be evaluated in this article, or claim that may be made by its manufacturer, is not guaranteed or endorsed by the publisher.
